# A Comprehensive Antimicrobial Activity Evaluation of the Recombinant Microcin J25 Against the Foodborne Pathogens *Salmonella* and *E. coli* O157:H7 by Using a Matrix of Conditions

**DOI:** 10.3389/fmicb.2019.01954

**Published:** 2019-08-27

**Authors:** Haitao Yu, Ning Li, Xiangfang Zeng, Lu Liu, Yuming Wang, Gang Wang, Shuang Cai, Shuo Huang, Xiuliang Ding, Qinglong Song, Shiyan Qiao

**Affiliations:** ^1^State Key Laboratory of Animal Nutrition, China Agricultural University, Beijing, China; ^2^Ministry of Agriculture and Rural Affairs Feed Industry Centre, China Agricultural University, Beijing, China; ^3^Beijing Key Laboratory of Bio-Feed Additives, Beijing, China

**Keywords:** *Salmonella*, *Escherichia coli* O157:H7, recombinant microcin J25, antimicrobial activity, biological environments, stability, mitigation

## Abstract

Natural microcin J25 (MccJ25) represent promising alternatives to traditional antibiotics for the treatment of drug-resistant infections. However, little is known about the antibacterial activity of recombinant MccJ25 against foodborne pathogens. Here, the activity of recombinant MccJ25 was examined using a matrix of conditions in order to assess the efficacy of recombinant MccJ25 as a mitigation against foodborne pathogens, such as *Salmonella* species and *Escherichia coli* (*E. coli*) O157:H7. Results showed that recombinant MccJ25 displayed excellent antimicrobial activity against these foodborne pathogens, including clinical isolates of *Salmonella* and *E. coli*, as well as clinical antibiotic-resistant *Salmonella* and *E. coli* isolates with different minimal inhibitory concentrations. In addition, antimicrobial activity curves and Live/Dead assay evidenced that recombinant MccJ25 harbors strong bactericidal activity against *Salmonella* and *E. coli* O157:H7. Notably, recombinant MccJ25 also had great potency and induced fast mortality against different growth phase of *Salmonella* and *E. coli.* The stability analysis results showed that the activity of recombinant MccJ25 was not influenced by temperatures as high as 121°C. Varying the pH from 2.0 to 9.0 did not appear to affect the activity of recombinant MccJ25. Under the challenge of several proteases, simulated gastrointestinal fluids and serum, recombinant MccJ25 still maintained exceptionally strong antimicrobial activity. Significant reductions in *Salmonella* Pullorum levels were also achieved in food biological environments, such as milk, egg and meat. Moreover, we demonstrated that recombinant MccJ25 appeared to act by inducing membrane breaks, thinning, and disintegration in the *Salmonella* Pullorum cytoplasmic membrane. Taken together, these results indicated that recombinant MccJ25 could be an effective alternative for mitigating and prevention of *Salmonella* and *E. coli* infection in food, animal and agriculture applications.

## Introduction

The most notorious and common pathogenic *Escherichia coli* (*E. coli*) O157:H7, *Salmonella* Typhimurium and *Salmonella* Pullorum strains are primary enteric pathogens that not only inhibit human and animal health but also cause food poisoning and food-related epidemics around the world (Sable et al., 2000; Ma et al., 2016; Fan et al., 2017). Especially, fresh produce, such as milk, meat, and egg, is increasingly related to the incidence of foodborne illness, and outbreaks associated with fresh produce consumption by human or animals have risen substantially recently (Centers for Disease Control and Prevention [CDC], 2010, 2013). As reported, from 1990 to 2005, around 800 outbreaks sickened over 34,000 people because of produce-associated foodborne illness (DeWaal and Bhuiya, 2007).

Contaminated food and water are possible sources of produce-related disease transmission because food and water resources can serve as natural reservoirs for *Salmonella* and *E. coli* (Black, 1990; Rajabi et al., 2011). It is estimated that over 50% of produce-related illnesses are caused by *Salmonella* and *E. coli*, which is the leading cause of both hospitalizations and deaths associated with bacterial foodborne illness (Centers for Disease Control and Prevention [CDC], 2011). In terms of controlling food or food processing water quality is needed to ensure the safety of human and animals. Commonly used antibiotics are now unsuccessful (Wlodarska et al., 2011; Cho et al., 2012; Rabanal et al., 2015) and are challenged by antibiotic-resistant pathogens (Moreno et al., 1995; Falagas et al., 2012; Penders et al., 2013). Therefore, there is an urgent need to encourage the produce industry to find more efficient and less toxic antimicrobial agents alternatives for treating bacterial infections, particularly those caused by foodborne pathogens, and to ensure human health, food safety, and animal health.

Nature AMP MccJ25 has attracted much attention as a potential agent for both humans and animals and as a food preservative due to its strong antimicrobial activity *in vitro* and *in vivo* (Black, 1990; Sassone-Corsi et al., 2016; Forkus et al., 2017). But, in recent years, chemical synthesis of AMPs is extreme costly and is not scalable for large-scale AMP production. Recombinant expression was always used to produce AMPs in many laboratories (Cao et al., 2018; Wei et al., 2018; Kim et al., 2019; Meng et al., 2019). Recombinant AMP MccJ25 can be large-scale produced by an MccJ25 expression vector and exhibit antimicrobial activity. For example, recombinant AMP MccJ25 exerted significant antimicrobial activity against *Salmonella, Shigella*, and *E. coli* (Yu et al., 2018a). Additionally, recombinant AMP MccJ25 was shown to effectively reduce *E. coli* in live piglets (Yu et al., 2017) and in healthy mice (Yu et al., 2018b). Recombinant AMP MccJ25 also did not cause toxic risk *in vivo* or *in vitro* (Yu et al., 2018a, b).

The effects of recombinant AMP MccJ25 on ETEC K88 were examined previously, but this research did not determine efficacy for different conditions and strains (Yu et al., 2018a). Additionally, the mode of action, antimicrobial properties in different biological or incubation conditions, and potential use of recombinant MccJ25 against *S.* Pullorum remains unclear. Therefore, it is critical for us to investigate the comprehensive antimicrobial activity of recombinant AMP in different environments. In the present study, we examined the anti-*Salmonella* and *E. coli* O157:H7 activity of recombinant AMP MccJ25 for various environmental conditions in order to evaluate its potential as an antimicrobial agent in food, agricultural, and animal practices. This assessment was performed to evaluate its potential as an antimicrobial agent for clinical studies and commercial use to treat bacterial infections and ensure food safety and animal health.

## Materials and Methods

### Preparation of Recombinant MccJ25

Recombinant MccJ25 was expressed and purified as has been described previously (Yu et al., 2018a), with minor modifications. A recombinant expression vector pMJ25 was constructed according to our lab standard protocol. Then, the recombinant expression vector was transformed into chemically competent *E. coli* BL21. The recombinant strain, *E. coli* BL21, was inoculated into a sucrose compound-based medium supplemented with the antibiotics kanamycin and ampicillin in 200 mL shaking flasks at 37°C at 200 rpm for 12 h. After expression, culture supernatants were harvested by centrifugation at 4°C and 12,000 rpm for 10 min. The recombinant MccJ25 was purified by HPLC, and the purity of the recombinant MccJ25 was greater than 99.95%. The recombinant MccJ25 concentrated stock solution was prepared in endotoxin-free water and stored at −20°C until use.

### Measurement of Minimum Inhibitory Concentration (MIC) and Minimum Bactericidal Concentration (MBC)

Four standard *Salmonella enterica* (*S. enterica*) serotype strains and *E. coli* O157:H7 are listed in Table 1 and were used to detect activity in Recombinant MccJ25 optimization studies. These strains were obtained from the China Institute of Veterinary Drug Control in Beijing. The recombinant MccJ25 sensitivity of the additional isolates was also examined for the strains listed in Table 2. These strains were obtained from intestinal contents of weaned pigs with diarrhea, diseased poultry and cattle (strains were provided by Beijing Bio-feed Additive Key Laboratory) as well as clinical sources (Dr. Wu Congming, College of Veterinary Medicine, China Agricultural University). The clinical source strains, referred to as CAU, were antibiotic (lincomycin and tilmicosin)-resistant *Salmonella* and *E. coli* isolates.

**TABLE 1:** Comparison of minimal inhibitory concentration (MIC) and minimum bactericidal concentration (MBC) of recombinant MccJ25 among *Salmonella* serotypes and *E. coli* O157:H7.

**Strains**	**MIC (μg/mL)^a^**	**MBC (μg/mL)^b^**
*Salmonella* pullorum CVCC1791	0.03	0.25
*Salmonella* pullorum CVCC534	8	8
*Salmonella* pullorum CVCC1811	8	8
*Salmonella* Typhimurium ATCC14028	16	4
*E. coli* O157:H7	16	8

*^*a*,*b*^All MIC and MBC values represent three independent experiments, each experiment was performed in triplicate.*

**TABLE 2:** MIC and MBC for recombinant MccJ25 with clinical strains.

**Strains**	**Source^a^**	**MIC (μg/mL)^b^**	**MBC (μg/mL)^c^**
*Salmonella*			
LKFZ SK225	Weaned pig stool	6.25	12.50
LKFZ SK226	Weaned pig stool	0.25	0.25
LKFZ SK232	Weaned pig stool	0.25	0.50
LKFZ SK236	Broiler chicken stool	0.50	2.00
LKFZ SK77	Broiler chicken stool	2.00	2.00
CAU QZL11	Dairy cow Mastitis	0.03	2.00
CAU QZL13	Dairy cow Mastitis	0.06	4.00
CAU QZL19	Dairy cow Mastitis	0.03	2.00

*^*a*^Strains named LKFZ were provided by Beijing Bio-feed Additive Key Laboratory. Clinical source strains designated CAU QZL were provide by Dr. Wu Congming, College of Veterinary Medicine, China Agricultural University). These clinical source strains were antibiotic (lincomycin and tilmicosin) resistant *Salmonella* and *E. coli*. ^*b*,*c*^All MIC and MBC values represent three independent experiments, each experiment was performed in triplicate.*

These strains were stored at −80°C in Luria broth (LB) and were prepared and autoclaved with 45% glycerol. For each experiment, *Salmonella* and *E. coli* strains were individually plated onto XLT4 and MacConkey agar (Beijing Land Bridge Technology Co., Beijing, China) for isolation. Single colonies were then inoculated into 10 mL of LB. Cultures were incubated at 37°C with overnight shaking (200 rpm) to reach a stationary phase with approximately 1 × 10^9^ colony-forming units (CFU)/mL. They were subsequently reinoculated into new test tubes with 10 mL of LB at 37°C for the indicated time to obtain 1 × 10^8^ CFU/mL log phase bacteria.

The MIC is described as the lowest concentration of the recombinant MccJ25 capable of preventing visible growth of the bacteria in susceptibility tests by broth dilution, as described previously (CLSI, 2012; Wei et al., 2018; Meng et al., 2019). The MBC is the first drug dilution with a 99.9% reduction in the initial bacterial concentration. MBC was determined in LB medium. Bacterial concentrations were adjusted to approximately 5 × 10^6^ CFU/mL. The concentrations of the recombinant MccJ25 ranged from 0.125 to 256 μg/mL. MIC and MBC were determined from three independent experiments, each experiment was performed in triplicate.

### Antimicrobial Activity Curves and Live/Dead Viability Assay

To further address the activity of recombinant MccJ25, antimicrobial activity curves and Live/Dead viability assay were performed. A single colony of *S.* Pullorum CVCC1791, *S.* Typhimurium ATCC14082, and *E. coli* O157:H7 was inoculated into 5 mL of LB. The culture was then incubated at 37°C with shaking at 200 rpm overnight. The following day, the culture was diluted 1:100 in fresh LB and again incubated at 37°C with shaking at 200 rpm. For antimicrobial activity, the bacteria were grown until they reached log phase. Approximately 5 × 10^4^ CFU/mL bacteria were inoculated into 2 mL of LB containing different concentrations of the recombinant MccJ25 (0, 1 × MIC, 2 × MIC, and 4 × MIC). The cultures (0.1 mL) were serially diluted with PBS and plated on LB and TSA agar at 0, 2, 4, and 6 h. The plates were incubated at 37°C overnight to count colony. Colonies were counted to determine CFU/mL. The experiment was conducted with two replicates in three independent trials.

A Live/Dead BacLight Bacterial Viability Kit 7 (Molecular Probes, Inc., Eugene) was used to test bacterial viability. Briefly, 5 × 10^4^ CFU/mL *S.* Pullorum, *S.* Typhimurium, and *E. coli* O157:H7 were inoculated into 1 mL of LB containing recombinant MccJ25 at the MIC level specific for each pathogen. Bacterial cultures were incubated at 37°C for 2 h and then incubated in the dark at ambient temperature with SYTO 9 and propidium iodide for 15 min. Bacteria were observed using a fluorescence microscope (EVOS XL Cell Imaging System).

### Antimicrobial Activity of Recombinant MccJ25 Against Pathogens in Different Growth Phases

The effect of growth phase on the anti-*Salmonella* and -*E. coli* O157:H7 activity of recombinant MccJ25 was determined by the viable plate counting method, as described previously (Ma et al., 2016; Fan et al., 2017), with minor modifications. Cultures of the strains in the early-log phase, late-log phase and stationary phase were washed, concentrated and diluted with sterilized phosphate-buffered saline (PBS) to approximately 10^7^ CFU/mL. Then, 1 mL was incubated individually in 9 mL of PBS to reach an approximate 10^6^ CFU/mL final concentration. The bacterial cells were then inoculated into 5 mL of LB containing different concentrations of MccJ25 (0, 1 × MIC, 2 × MIC, and 4 × MIC). At 0, 12, 24, and 48 h post incubation, the cultures (0.1 mL) were serially diluted with PBS and plated on LB and TSA agar. The plates were incubated at 37°C overnight to count colony. Colonies were counted to determine CFU/mL. The experiment was conducted with two replicates in three independent trials.

### Antimicrobial Activity of Recombinant MccJ25 Against Pathogens in Biological Products

To further investigate the effects of recombinant MccJ25 on *Salmonella* and *E. coli* O157:H7 in different biological food products, sterile skim milk, egg yolk and mincemeat (pork) extract were collected and used as described previously with minor modifications (Sable et al., 2000). The skim milk, egg and mincemeat samples were purchased from a Carrefour store in Beijing. Specimens were kept on ice packs in coolers until they reached the laboratory, where they were stored at 4°C. The skim milk (SKM) and mincemeat supernatant (SMS) were autoclave sterilized at 121°C for 20 min. After autoclaving, the SKM and SMS samples were stored at 4°C for further use. The eggs were sterilized with 75% ethanol and transferred into an exceptionally clean bench to collect the egg yolk (SEY). The SEY was diluted 1:1 with sterile distilled water. Biological product samples were mixed with a final concentration of 4 × MIC of recombinant MccJ25 solution in sterile saline water (0.85%). They were then inoculated with *S.* Pullorum, *S.* Typhimurium and *E. coli* O157:H7 at a final concentration of 10^6^ CFU/mL. Sterilized saline water (SSW) with the recombinant MccJ25 was utilized for comparison. This assay was performed to obtain the desired quantity of cells. The mixtures were tested after 24 h of incubation at 37°C with shaking (250 rpm). After incubation, *Salmonella species* and *E. coli* O157:H7 were serially diluted with PBS and plated on LB and TSA agar. The plates were incubated at 37°C overnight to count colony. Colonies were counted to determine CFU/mL. The experiment was conducted with two replicates in three independent trials.

### The Effect of Temperature and pH on the Anti-*Salmonella* Activity of Recombinant MccJ25

Assays of the antibacterial activity of recombinant MccJ25 at different temperatures (25, 37, 45, 55, 65, 75, 85, and 95°C for 20 min) and different pH values (pH 2.0, 3.0, 4.0, 5.0, 6.0, 7.0, 8.0, and 9.0 for 2 h) were performed. The assays were conducted as described previously (Chen et al., 2009; Meng et al., 2019), with slight modifications. LB agar inoculated with the indicator bacteria *S.* Pullorum (approximately 10^5^ viable cells per mL) was poured over plates fitted with Oxford cups to make wells. 150 μL of 1 × MIC final concentration of recombinant MccJ25 samples previously treated under various conditions were added to the wells. The agar overlays were incubated overnight at 37°C, and the inhibition zones were recorded.

### Effect of a Single Protease on the Antimicrobial Activity of Recombinant MccJ25 Against *Salmonella*

The resistance of recombinant MccJ25 to pepsin, trypsin and chymotrypsin was evaluated as described previously (Chen et al., 2009; Meng et al., 2019) with slight modification. The indicator *Salmonella* was prepared (approximately 1 × 10^7^ CFU/mL), and 1 mL of bacterial inoculum was transferred into 9 mL of each medium with the recombinant MccJ25 and into corresponding control media without the recombinant MccJ25. LB agar inoculated with the indicator bacteria *S.* Pullorum (approximately 10^5^ viable cells per mL) was poured over plates that had been fitted with Oxford cups to make wells. Then, 150 μL of a 1 × MIC final concentration of recombinant MccJ25 that had been previously treated under various conditions for 2 and 4 h were added to the wells. The agar overlays were incubated overnight at 37°C, and the inhibition zones were recorded.

### Determination of Antimicrobial Activity of Recombinant MccJ25 in Biological Conditions

Biological fluids (SGF; SIF) and serum were prepared as described previously (Liu et al., 2013; Xia et al., 2015). Then, assays of the antibacterial activity of recombinant MccJ25 in SGF, SIF and serum were performed. *S.* Pullorum was grown in Mueller-Hinton broth (MHB, Difco Laboratories, Detroit, MI, United States). One milliliter of bacterial inoculum was transferred into 9 mL of each medium with the recombinant MccJ25 and into corresponding control medium without the recombinant MccJ25. LB agar inoculated with the indicator bacteria *S.* Pullorum (approximately 10^5^ viable cells per mL) was poured over plates fitted with Oxford cups to make wells. Hundred and fifty μL of a 1 × MIC final concentration of recombinant MccJ25 samples that had previously been treated with SGF, SIF and serum for 2, 4, and 6 h, respectively, were added to the wells. The agar overlays were incubated overnight at 37°C, and the inhibition zones were recorded.

### Anti-*Salmonella* Mechanism Study

To assess the alteration of *S.* Pullorum cell membrane morphology and ultrastructure, SEM and TEM were performed as described previously, with minor modifications (Wang et al., 2017; Meng et al., 2019). For SEM sample preparation, approximately 2 × 10^6^ *S.* Pullorum cells were incubated at 37°C for 1 h with the recombinant MccJ25 at 1× and 4 × MIC levels. The control was performed without the recombinant MccJ25. After incubation, the cells were harvested, mixed and centrifuged at 8,000 × *g* for 3 min. The bacterial pellets were washed three times in PBS and centrifuged at 3,000 × *g* for 10 min. The bacterial cells were resuspended in 500 μL of PBS and supplemented with 2.5% fresh glutaraldehyde at 4°C overnight. Thereafter, the bacterial pellets were postfixed in 1% osmium tetroxide, dehydrated, and coated with gold-palladium. They were observed by SEM (EVO MA10 XVP, Carl Zeiss).

For TEM sample preparation, (1) two milliliters of 2 × 10^6^ cells of *S.* Pullorum broth cultured in the absence or presence of 1×, 2× and 4× MIC of the recombinant MccJ25 were incubated and collected at 1 h. (2) After that, approximately 2 × 10^6^ *S.* Pullorum cells were incubated at 37°C for 1 h with the recombinant MccJ25 at 2 × MIC for 2, 3, and 4 h. They were then centrifuged at 5,000 × *g* for 5 min. Pretreatment of bacterial samples was conducted in the same way as the SEM treatment. Finally, the samples were placed in capsules containing embedding medium and heated at 70°C for approximately 9 h. Specimens were stained with uranyl acetate and alkaline lead citrate for 15 min. They were then observed by TEM (Model JEM-1230, JEOL, Japan).

### Statistical Analysis

Microbiological population results were log transformed for statistical analysis. The results are expressed as the mean ± standard error of the mean (SEM). Data were analyzed by one-way ANOVA with the use of GraphPad Prism 6 (GraphPad Software Inc., San Diego, CA, United States). Tukey’s *post hoc* test was applied to determine differences between treatment means. All data were visualized using GraphPad Prism 6. Statistical significance was expressed using *P* < 0.05.

## Results

### Variability in Recombinant MccJ25 Sensitivity Among *Salmonella* and *E. coli* Strains

As shown in Table 1, recombinant MccJ25 activity was tested for four standard *Salmonella* strains, including the serotypes Pullorum (*n* = 3) and Typhimurium (*n* = 1), and for *E. coli* O157:H7. The results are shown by MIC and MBC. Recombinant MccJ25 was effective and showed the lowest MIC (0.03 μg/mL) and MBC (0.25 μg/mL) against *S.* Pullorum CVCC1791. In comparison, the MICs and MBCs of recombinant MccJ25 against *S.* Pullorum CVCC534, *S.* Pullorum CVCC1811, *S.* Typhimurium ATCC14028 and *E. coli* O157:H7 were 8/8, 8/8, 16/4 and 16/8 μg/mL, respectively. Additionally, all pathogens treated with recombinant MccJ25 at various MBC levels (Table 1) were killed. Bactericidal activity is presented as MBC and is defined as eliminating 99.9% of the bacteria of the tested species.

Similar to foodborne *E. coli* O157:H7, *Salmonella* is one of the most common pathogens associated with foodborne disease worldwide. *Salmonella* strains included isolates from clinical sources (*n* = 8) and are presented in Table 2. Recombinant MccJ25 showed strong antimicrobial activity (low MIC and MBC) against these clinically relevant *Salmonella* strains. For bactericidal activity, the results showed that most of the pathogens treated with recombinant MccJ25 at different MBC levels (Table 2) were killed. These assays indicated that the recombinant MccJ25 exerted antibacterial and bactericidal activity against the foodborne pathogens *Salmonella* and *E. coli* O157:H7.

### Antimicrobial Activity of Recombinant MccJ25

**FIGURE 1:**
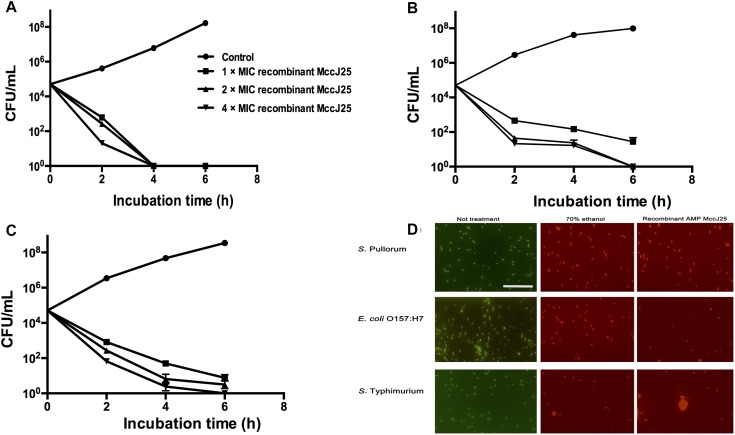
Recombinant MccJ25 kills foodborne pathogens. Killing curves of *S.* Pullorum **(A)**, *E. coli* O157:H7 **(B)**, and *S.* Typhimurium **(C)** for different concentrations of recombinant MccJ25. Survival of bacteria was compared at 0, 2, 4, and 6 h post inoculation. Viable cells were determined by plate counts, and mean CFU/mL ± SEMs is shown for three individual experiments. *n* = 6. **(D)** Fluorescent micrograph of cells treated with 0% recombinant MccJ25 (left), 70% ethanol (middle), or recombinant MccJ25 at the MIC level (right). The white bar indicates 20 μm. The results shown are representative of three independent experiments.

To clarify the effectiveness of the antimicrobial activity of recombinant MccJ25, we performed a time-course antimicrobial assay. Killing curves for pathogenic *S.* Pullorum (Figure 1A), *S.* Typhimurium (Figure 1B), and *E. coli* O157:H7 (Figure 1C) in the presence of recombinant MccJ25 were determined. These pathogens were collected every 2 h during incubation to count viable cells for 6 h. Recombinant MccJ25 eliminated the above pathogens after 6 h of incubation when a final concentration of 4 × MIC was applied (Figure 1). However, for the pathogen *S.* Pullorum (Figure 1A), when the concentration was lowered to 1 × MIC, recombinant MccJ25 killed all bacteria after 4 h and did not exhibit regrowth after 8 h of incubation. Therefore, the Killing curve data confirmed that recombinant MccJ25 has a stronger antimicrobial activity against *S.* Pullorum than against *S.* Typhimurium and *E. coli* O157:H7.

Additionally, to further investigate the bactericidal activity of recombinant MccJ25 against *S.* Pullorum, *S.* Typhimurium and *E. coli* O157:H7, we conducted a Live/Dead antimicrobial assay. As shown in Figure 1D, live bacteria without treatment emit green fluorescence, which are stained with SYTO 9, while dead bacteria treated with 70% ethanol treatment emit red fluorescence, which are stained with propidium iodide, as this dye penetrates into the cytosol through the damaged membranes. Recombinant MccJ25 had excellent bactericidal activity against all challenging microorganisms tested.

### The Effects of Recombinant MccJ25 on Different Phase Cultures of *Salmonella* and *E. coli* O157:H7

**FIGURE 2:**
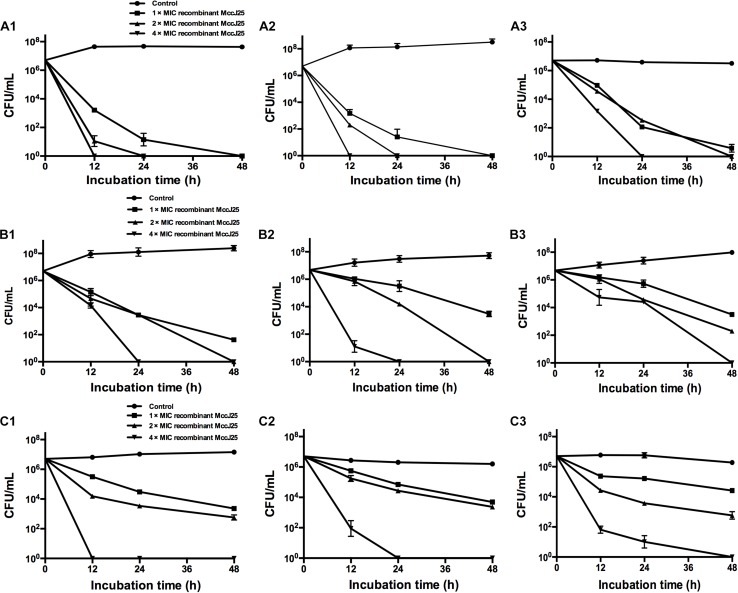
Recombinant MccJ25 exerts strong antimicrobial activity against different growth phase foodborne pathogens. *S.* Pullorum **(A1–A3)**, *S.* Typhimurium **(B1–B3)**, and *E. coli* O157:H7 **(C1–C3)** were grown to early-log **(A1, B1, C1)**, late-log **(A2, B2, C2)**, and stationary **(A3, B3, C3)** phases. Then, approximately 10^7^ CFU/mL bacteria were inoculated into LB medium containing different concentrations of recombinant MccJ25. Survival of strains was compared at 0, 12, 24, and 48 h post inoculation. *Salmonella* and *E. coli* levels (mean CFU/mL ± SEMs) were determined from three individual experiments. *n* = 6.

The antimicrobial activity of a range of recombinant MccJ25 concentrations against *Salmonella* and *E. coli* O157:H7 was examined at 12, 24, and 48 h at different growth phases, including the early-log phase, late-log phase and stationary phase. Regardless of the bacterial growth phase, recombinant MccJ25 exhibited significant antimicrobial activity against *S.* Pullorum, *S.* Typhimurium, and *E. coli* O157:H7 (Figure 2). *S.* Pullorum cells at early-log (Figure 2A1), late-log (Figure 2A2), and stationary phase (Figure 2A3) were effectively killed by recombinant MccJ25. Early- or late-log phase cells of *S.* Pullorum were reduced to undetectable levels within 24 h when treated with 8 μg/mL recombinant MccJ25 and by 12 h when treated with 4 × MIC of recombinant MccJ25 (Figures 2A1,A2). Moreover, there was a reduction in stationary phase cells of *S.* Pullorum to non-detectable levels within 24 h at concentrations of 16 μg/mL recombinant MccJ25 and by 48 h with 8 μg/mL recombinant MccJ25.

When the early and late-log phase cells of *S.* Typhimurium were incubated with recombinant MccJ25 at various concentrations, *S.* Typhimurium was killed within 24 h at 4 × MIC recombinant MccJ25 and by 48 h with 2 × MIC recombinant MccJ25 (Figures 2B1,B2). However, *S.* Typhimurium exhibited significantly greater resistance to recombinant MccJ25 in the stationary phase than in the log phase. Additionally, reduction to non-detectable levels required 4 × MIC recombinant MccJ25 at 48 h (Figure 2B3). However, a reduction in early- and late-log phase *E. coli* O157:H7 to non-detectable levels within 12 and 24 h, respectively, required a concentration of 4 × MIC recombinant MccJ25 (Figures 2C1,C2). Bacteria exhibited significantly greater resistance to recombinant MccJ25 in the stationary phase than in the early-log and late-log phases, as well as a reduction in non-detectable levels requiring 4 × MIC recombinant MccJ25 and 48 h (Figure 2C3). Although the antimicrobial activity decreased in the different phases for the different strains, recombinant MccJ25 still exerted immense antimicrobial activity against all pathogens.

### Recombinant MccJ25’s Bactericidal Capacity in Biological Products

**FIGURE 3:**
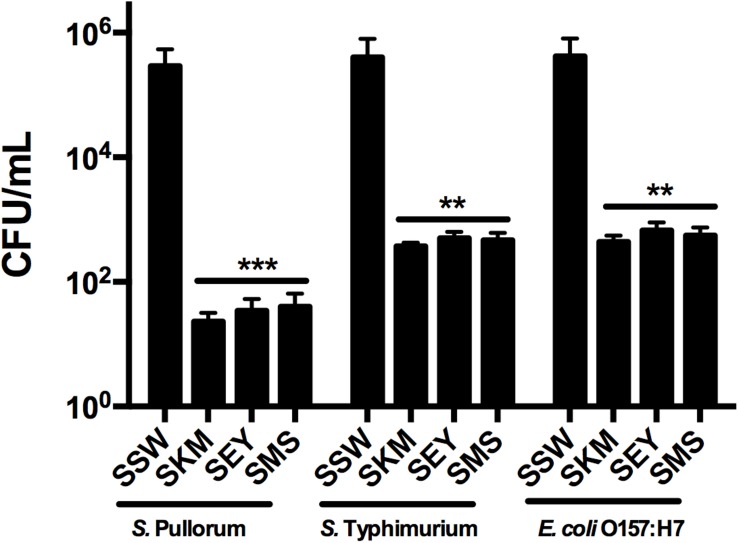
Antimicrobial activity of recombinant MccJ25 against foodborne pathogens in biological products. Approximately 5 × 10^6^ CFU/mL bacteria were inoculated into autoclaved skim milk (SKM), mincemeat supernatant (SMS), and sterilized egg yolk (SEY). CFU Reduction in bacteria after 24 h in SKM, SMS, and SEY containing 4 × MIC recombinant MccJ25. Viable cells were determined by plate counts, and mean CFU/mL ± SEMs is shown for three individual experiments. *n* = 6. The asterisk denotes a significant difference in comparison with the control group (^∗∗^*P* < 0.01, ^∗∗∗^*P* < 0.001).

The efficacy of recombinant MccJ25 against inoculated *Salmonella* and *E. coli* O157:H7 in different food samples was tested. When the three products contained 4 × MIC recombinant MccJ25 and all pathogen concentrations were 10^3^ CFU/mL, no viable bacteria were detected after 24 h (0 CFU in three 100 μL product samples, data not shown). With all pathogenic bacteria concentrations at 10^4^ CFU/mL, we obtained the same results (no bacteria were detected, data not shown) for the three products. However, when the concentration of all pathogens was 10^6^ CFU/mL, *Salmonella* and *E. coli* O157:H7 cultures inoculated into the three biological products (SKM, SEY, and SMS) with 4 × MIC recombinant MccJ25 decreased significantly. *S.* Pullorum reductions of approximately 10^4^, 8 × 10^3^, and 7 × 10^3^ CFU/mL were achieved for SKM, SEY, and SMS, respectively. Furthermore, an approximately 10^3^ CFU/mL reduction in *S.* Typhimurium and *E. coli* O157:H7 was achieved for SKM, SEY, and SMS (Figure 3).

### Overall Evaluation of All Incubation Biological and Biochemical Parameters

**FIGURE 4:**
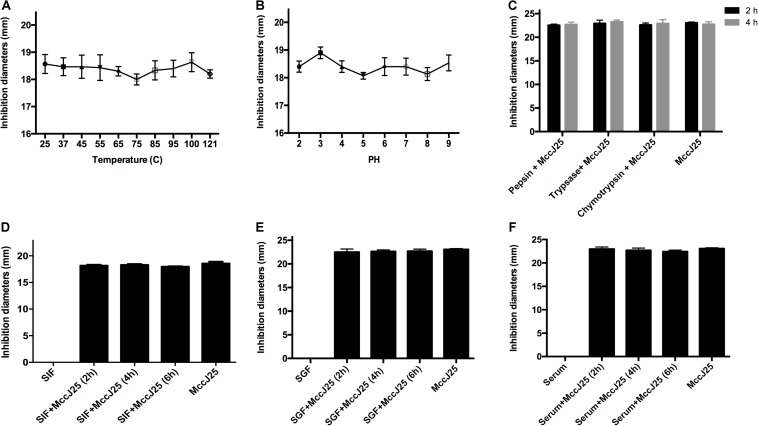
Effects of temperature **(A)**, pH **(B)**, and proteases **(C)** on inhibition of *Salmonella* by recombinant MccJ25, as well as antimicrobial activity of recombinant MccJ25 against *Salmonella* in the presence of SGF **(D)**, SIF **(E)**, and serum **(F)**. The inhibition zone method was used to assess the antimicrobial activity of recombinant MccJ25. The graphs were derived from average values for three replicate experiments and almost identical triplicate sets of data. No significant difference was detected among treatments (*P* > 0.05).

To further investigate whether recombinant MccJ25 maintained antimicrobial activity *in vivo*, an *S.* Pullorum strain was used as the indicator pathogen because the data presented in the above experiments showed stronger antimicrobial activity against *S.* Pullorum than against either *E. coli* O157:H7 or *S.* Typhimurium. The bactericidal potency of recombinant MccJ25 for *S.* Pullorum was tested under different conditions, as summarized in Figures 4A–F. The results of the thermal stability test showed that temperatures as high as 121°C for 20 min had no influence on the effect of recombinant MccJ25 against *S.* Pullorum (Figure 4A). Consistent with the thermal stability results, varying the pH from 2.0 to 9.0 also had no effect on the effect on recombinant MccJ25 activity against *S.* Pullorum (Figure 4B). Moreover, under the challenge of three proteases (pepsin, trypsin, and chymotrypsin) for 2 and 4 h, recombinant MccJ25 maintained its antimicrobial activity, as shown in Figure 4C.

To further assess the therapeutic potential of recombinant MccJ25, we first assayed its effect in the presence of simulated gastrointestinal fluids and serum. When incubated with SGF (Figure 4D), SIF (Figure 4E) or serum (Figure 4F), recombinant MccJ25 still inhibited the growth of *S.* Pullorum in a manner similar to that of recombinant MccJ25 alone. These results suggest that recombinant MccJ25 is stable in the gastrointestinal tract and remains effective.

### Disruption of Bacterial Membrane Integrity

**FIGURE 5:**
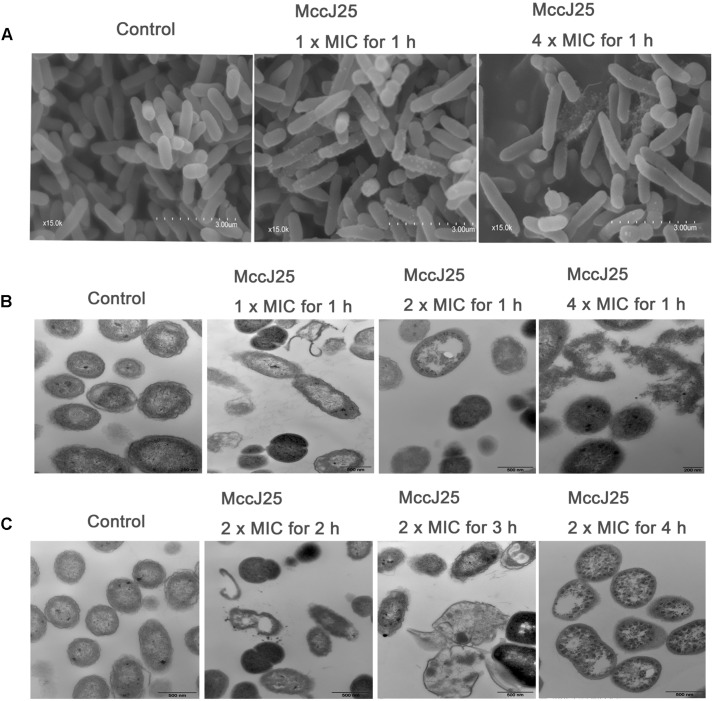
SEM **(A)** and TEM **(B,C)** micrographs of *S.* Pullorum. For SEM, bacteria were treated with recombinant MccJ25 at 1 and 4 × MIC for 1 h. The scale bar is 3 μm. For TEM, bacteria were treated with recombinant MccJ25 at 2 × MIC for 2, 3, and 4 h. The control was performed without recombinant MccJ25. The scale bar is 0.5 μm. Data are the means ± SEMs of three independent experiments.

SEM and TEM have been used to directly observe the effects of AMPs on the cell morphology and integrity of bacteria. In the present study, the microbicidal properties of recombinant MccJ25 were measured by observing the morphology and integrity of the *S.* Pullorum cell membrane. As shown in Figure 5, untreated cells in the control group had a smooth and bright surface as well as intact cell morphology. Treatment with 1 or 4 × MIC recombinant MccJ25 for 1 h, which did not inhibit the growth of *S.* Pullorum, resulted in minimal membrane roughening, enlargement, and some filiferous adhesions in a small amount of cells (Figure 5A).

TEM was also used to explore the morphology and intracellular alteration of the bacteria (Figures 5B,C). The results showed that the integrity of the cytoplasmic membrane of *S.* Pullorum after treatment with 1, 2, or 4 × MIC recombinant MccJ25 for 1 h was adversely affected (Figure 5B). Compared to the control, recombinant MccJ25 significantly disrupted the membrane of *S.* Pullorum, and some cytoplasmic clear zones were visible. Leakage of intracellular contents was also observed in recombinant MccJ25-treated *S.* Pullorum cells. However, 2 × MIC recombinant MccJ25 treatment of bacteria for different times (2, 3, and 4 h) showed deformed small septa, invisible cytoplasmic membranes, heterogeneous electron density, electron-light regions and thinned cell walls. Total leakage of cytoplasmic contents was also evident (Figure 5C).

## Discussion

The number of foodborne pathogens has increased rapidly in recent years, especially those of *Salmonella* and *E. coli* species (Ma et al., 2016; Fan et al., 2017; Yi et al., 2017). Current treatments are limited due to the loss of antimicrobial agent efficacy and the slow development of new drugs and antibiotics (Ling et al., 2015; Rabanal et al., 2015; Yi et al., 2017). Practical solutions for these pathogens in food, animals and humans are not available (Hancock and Sahl, 2006). Therefore, the development of alternative antimicrobial agents for human health, food safety, and animal health is of critical importance. Nowadays, recombinant AMPs is promising alternatives to conventional antibiotics for the treatment of pathogens infections because of chemical synthesis is not suited for synthesis of large AMPs and is not scalable for large-scale production for clinical studies and commercial use (Cao et al., 2018; Wei et al., 2018; Kim et al., 2019; Meng et al., 2019).

The effects of recombinant MccJ25 on survival and growth of *Salmonella* and *E. coli* in different environments were examined in order to ascertain its potential as a antimicrobial agent for various domains applications. The results of assaying the antimicrobial activity of recombinant MccJ25 showed that sensitivity to the effects of recombinant MccJ25 varied among the different *Salmonella* strains and was not related to serotype. This finding is because differences were observed within strains of the same Pullorum serotype. Among all the tested bacteria, recombinant MccJ25 had the lowest MIC and MBC against *S.* Pullorum. These results corroborate our previous study. This finding indicates that recombinant MccJ25 inhibits *S.* Pullorum, as determined by an inhibition zone assay (Yu et al., 2018a). However, recombinant MccJ25 inhibits pathogenic bacteria *E. coli* O157:H7 and *S.* Typhimurium with an MIC of 16 μg/mL. This result could be directly attributed to the mechanism of action of MccJ25. This finding is consistent with previous studies that have found that MccJ25 has different mechanisms of action against different gram-negative bacteria and the same gram-negative bacteria at different MICs (Rintoul et al., 2001; Mukhopadhyay et al., 2004; Bellomio et al., 2007; Chirou et al., 2008). Additionally, previous reports have shown that microcins and recombinant AMPs exhibit different antimicrobial features and activity against gram-negative bacteria (Thomas et al., 2004; Severinov et al., 2007; Wei et al., 2018; Meng et al., 2019).

To further understand the effectiveness of the antimicrobial effect of recombinant MccJ25, we conducted a time-course and Live/Dead antimicrobial assay. The effects of recombinant MccJ25 on the survival and growth of *S.* Pullorum, *S.* Typhimurium and *E. coli* O157:H7 were examined to ascertain its potential as an antimicrobial agent and bioactive compound for food, animal or agricultural applications. The antimicrobial activity of recombinant MccJ25 was concentration dependent, as antimicrobial activity increased when more recombinant MccJ25 was added to the cultures. Our results corroborate previous studies indicating that recombinant MccJ25 has a different spectrum of activity against pathogens. They also showed that bacteria had varying sensitivities to the microcins (Hancock and Sahl, 2006; Ling et al., 2015). Additionally, Han et al. (2011) have shown that six different AMPs derived from five different animal species have different time curves for *E. coli* mortality. In addition, recombinant MccJ25 breaks bacterial cell walls, as shown by Live/Dead assays staining with propidium iodide, suggesting that recombinant MccJ25 may have a mode of action to kill pathogens similar to that of other AMPs or nano/microparticles (Han et al., 2011; Ling et al., 2015; Ma et al., 2016).

Some studies have shown that the antimicrobial activity of antimicrobial agents is unrelated to bacterial cell wall synthesis; however, they do disrupt bacterial cell walls (Ma et al., 2016; Fan et al., 2017). To examine this hypothesis, we employed bacterial cells at different growth phases for evaluating bactericidal activity. Although *Salmonella* strains and *E. coli* O157:H7 varied in their sensitivity, different concentrations of recombinant MccJ25 eliminated log phase (early- and late-log) bacteria for all strains examined at different indicated hours. Compared with log phase pathogens, stationary phase cells were more resistant to recombinant MccJ25, yet dramatic reductions were still achieved. It is known that stationary phase bacteria are more resistant than log phase cells to various antimicrobial treatments, including high pressure, heat, and biocides (Lee et al., 1994; Tabak et al., 2007; Cherchi and Gu, 2011). Manas and Mackey (2004) have also shown that stationary phase bacteria are stable because of intact outer membranes with higher protein and lipid ratios, lower unsaturated fatty acid content, and thicker cell walls than those of log phase bacteria. Although bacteria in food, the gut or water may be in the stationary phase due to nutrient limitations and stress, a reduction in *Salmonella* and *E. coli* O157:H7 cells in the stationary phase would still be feasible at high concentrations of recombinant MccJ25 or with long incubation times.

Thus, we carried out further studies of recombinant MccJ25 growth-inhibiting properties based on the above experiments on different biological food products and incubation conditions. In Sable et al.’s (2000) study, purified MccJ25 completely inhibition of the growth of 10^4^ CFU/mL *E. coli* O157:H7 required a concentration of 50 μg/mL in egg yolk. This result was because of the richness of the egg yolk medium. However, in our study, we found that 4 × MIC recombinant MccJ25 could halt the growth of 10^3^ and 10^4^ CFU/mL *Salmonella* and *E. coli* O157:H7 in different food products. Furthermore, in the rich egg yolk medium, 4 × MIC recombinant MccJ25 could halt the growth of *E. coli* O157:H7 and *Salmonella*. The reason for this discrepancy may be a possible variation in the purification approach, the purity of MccJ25, or the design of the expression vector. Moreover, when the concentration of *Salmonella* and *E. coli* O157:H7 reached 10^6^ CFU/mL in food products, 4 × MIC recombinant MccJ25 did not halt the growth of bacteria but did significantly reduce their number. Recombinant MccJ25 exerted stronger antimicrobial activity for the target *Salmonella* strain in the three food products than for *E. coli* O157:H7.

The data presented in the above experiments reveal the possibility of using recombinant MccJ25 to control pathogens. As we know, for any application of antimicrobial agents in food, humans or livestock, it is critical to identify any factors that could interfere with its activity, such as temperature, pH or proteases (Chen et al., 2009; Liu et al., 2013). A major limitation of AMPs is their low stability toward proteases (Chen et al., 2009; Han et al., 2011; Liu et al., 2013). This characteristic limits the practical usage of many peptides as drugs or food additives for both animals and humans. In the present study, the antimicrobial activity of recombinant MccJ25 against *S.* Pullorum was retained after varying temperature, pH, pepsin, trypsin and chymotrypsin treatments. These findings are consistent with prior reports that indicate MccJ25 is stable in complex conditions (Rosengren et al., 2003; Rebuffat et al., 2004). Additionally, the *in vitro* antimicrobial activity of recombinant MccJ25 was evaluated in simulated gastrointestinal environments and serum. This assessment was done to understand the fate of recombinant MccJ25 when it is orally administered. Our study reveals that recombinant MccJ25 exerts antibacterial activity in murine serum, in the stomach and in the intestinal tract. This observation is consistent with previous reports that some AMPs or micro-nano biomaterials have a stable bactericidal effect that is retained in SGF, SIF and serum (Liu et al., 2013; Xia et al., 2015; Ma et al., 2016).

We further confirmed the antimicrobial activities of recombinant MccJ25 by SEM and TEM. In our study, we found that recombinant MccJ25 has an acute negative effect on inner membrane integrity. As observed by TEM, the cytoplasmic membrane thinned, broke, and disintegrated. Abnormal separation was also observed. To our knowledge, this study was the first time that recombinant MccJ25 was observed to lead to lysis of the *S.* Pullorum cytoplasmic membrane and release of fluid and debris. Previous published studies have reported that MccJ25 disrupts membrane integrity in *S. enterica* Newport, in liposomes, and in uncharged phospholipid monolayers. However, these properties have been reported to be specific to *S. enterica* serovars and have been observed at concentrations greater than those of the MICs (Rintoul et al., 2001). Overall, our findings are consistent with an acting mechanism confined to the cytoplasmic membrane. These macroscopic effects indicate that these peptides might kill cells in different ways. Moreover, most AMPs disrupt membranes or induce pore/ion channel formation in the cytoplasmic membranes of susceptible bacteria (Han et al., 2011; Li et al., 2017; Wang et al., 2017). Additionally, some AMPs have multiple potential targets in bacteria, including the DNA, RNA, cell wall, cytoplasmic membrane, and various enzymes (Lee et al., 2016). On the other hand, our previous study has been shown that recombinant MccJ25 suppress intestinal inflammation and improve intestinal epithelial barrier function independently of their antimicrobial activity (Yu et al., 2018a). Consistent with the previous study, in the present study, we also found that recombinant MccJ25 inhibited *S.* Pullorum invasion and attenuated *S.* Pullorum-induced decreases in TEER and increases in FD4 and LDH levels (Supplementary Figure S2). To further investigate, the antibacterial activity of recombinant MccJ25 treatments on IPEC-J2 cells against *S.* Pullorum may be attributed to tight junction proteins (TJPs) expression (Supplementary Figures S1, S3). These results provide further evidence that recombinant MccJ25 is a candidate for clinical therapeutic use. For the determination of TJPs expression, the primers used are listed in Supplementary Table S1.

To summarize, this work showed that recombinant MccJ25 exhibits the greatest antimicrobial activity against different phases of *Salmonella* and *E. coli*, as well as harbors strong antimicrobial activity in various environments, thus mimicking real-world situations. In particular, examining the activity of recombinant MccJ25 against *Salmonella* and *E. coli* O157:H7 in food products showed the potential of recombinant MccJ25 as an antimicrobial agent or bioactive compound in the food industry. Recombinant MccJ25 has advantages over toxic chemicals commonly used for food and animal application due to its lack of toxicity, high bio-degradability, cost, availability, and reduced public health concerns. Although we identified relatively resistant strains of *Salmonella* and *E. coli*, high efficacy of recombinant MccJ25 (MIC was less or equal to 6.25 μg/mL) was found for all strains. Overall, the results of our study show great potential of recombinant MccJ25 to treat pathogens infections diseases caused by *Salmonella* and *E. coli*, especially multidrug resistant microorganisms, without adverse side effects. These data also support further investigations for larger scale application of a recombinant MccJ25 that offers broad range antimicrobial activity and low environmental impact.

## Author Contributions

SQ, XZ, and QS obtained the financial support. SQ, XZ, and HY designed and oversaw the study. HY, NL, and LL performed the cells experiment and cultured the cells. HY, SC, and YW conducted and performed the qPCR. HY, GW, and SH analyzed the data. XD provided the recombinant MccJ25 sample. HY wrote the manuscript with contributions from all other authors. All authors read and approved the final manuscript. SQ is the guarantor of this work.

## Conflict of Interest Statement

The authors declare that the research was conducted in the absence of any commercial or financial relationships that could be construed as a potential conflict of interest.

## Abbreviations

AMPs: antimicrobial peptides
*E. coli*: *Escherichia coli*
ETEC: enterotoxigenic *E. coli*
MBC: minimum bactericidal concentration
MccJ25: microcin J25
MIC: minimum inhibitory concentration
PBS: phosphate buffered saline
SEM: scanning electron microscopy
SEY: sterilized egg yolk
SGF: simulated gastric fluid
SIF: simulated intestinal fluid
SKM: sterilized skim milk
SMS: sterilized mincemeat supernatant
*S.* Pullorum: *Salmonella* Pullorum
*S. Typhimurium*: *Salmonella* Typhimurium
TEM: transmission electron microscopy
TSA: Trypticase Soy agar
XLT4: xylose lysine tergitol 4.
