# Supplementary material for: A Comprehensive Antimicrobial Activity Evaluation of the Recombinant Microcin J25 Against the Foodborne Pathogens Salmonella and E. coli O157:H7 by Using a Matrix of Conditions
Source: Front Microbiol. 2019 Aug 27;10:1954. doi: 10.3389/fmicb.2019.01954 (PMC6718478; doi:10.3389/fmicb.2019.01954)
Supplement: Supplementary file 1 [file Data_Sheet_1.docx]

**Supplementary Information**

**A Comprehensive Antimicrobial Activity Evaluation of the Recombinant Microcin J25 Against the Foodborne Pathogens *Salmonella* and *E. coli* O157 by using a matrix of conditions**

***Haitao Yu^1,2,3^, Ning Li ^1,2,3^, Xiangfang Zeng ^1,2,3^, Lu Liu ^1,2,3^, Yuming Wang^1,2,3^, Gang Wang ^1,2,3^, Shuang Cai ^1,2,3^, Shuo Huang^1,2,3^, Xiuliang Ding^1,2,3^, Qinglong Song^1,2,3^, and Shiyan Qiao^1,2,3*^***

**FIGURE S1** **|** Protective effects of recombinant MccJ25 against *Salmonella* invasion of IPEC-J2 cell monolayers. IPEC-J2 cells were cultured with or without recombinant MccJ25 for 6 h in the absence or presence of *Salmonella* for 2 h. The invasion of *Salmonella* in the IPEC-J2 cells was determined. *Salmonella* (mean CFU/ml ± SEMs) were determined by plate counts, and determined from three individual experiments. *n* = 9. Different superscript lowercase letters within each group indicate significant differences (P < 0.05).

**FIGURE S2** **|** Recombinant MccJ25 inhibited *Salmonella*-induced damage to IPEC-J2 cells. IPEC-J2 cells were cultured with or without recombinant MccJ25 (2 μg/mL) for 6 h in the absence or presence of *Salmonella* for 2 h. TEER **(A)**, FD4 level **(B)**, and LDH activity **(C)** in the IPEC-J2 cells were determined. Data are the means ± SEMs of three independent experiments, *n* = 9. Different superscript lowercase letters within each group indicate significant differences (*P* < 0.05); The asterisk denotes a significant difference in comparison with the control group (**P* < 0.05, ***P* < 0.01).

**FIGURE S3** **|** Recombinant MccJ25 significantly increased the mRNA expression of the tight junction proteins ZO-1 and Claudin-1 in *Salmonella*-challenged IPEC-J2 cells. IPEC-J2 cells were cultured with or without recombinant MccJ25 for 6 h and then treated with *Salmonella* for 2 h. Cells were collected, and relative mRNA expression was analyzed by real-time PCR. The data are the means ± SEMs of three independent experiments, *n* = 9. Different superscript lowercase letters within each group indicate significant differences (*P* < 0.05).

**TABLE S1 |** Sequences of oligonucleotide primers for gene expression using real-time PCR.

| Gene | Primer Sequence | Product Size | GeneBank Accession No. |
| --- | --- | --- | --- |
| *β*-actin | F:5'-TGCGGGACATCAAGGAGAAG-3'  R: 5'-AGTTGAAGGTGGTCTCGTGG-3' | 217 | DQ845171 |
| ZO-1 | F: 5'-AAGGATGTTTACCGTCGCATT-3'  R: 5'-ATTGGACACTGGCTAACTGCT-3' | 253 | XM_003353439.2 |
| claudin-1 | F: 5'-GCTGGGTTTCATCCTGGCTTCT-3'  R: 5'-CCTGAGCGGTCACGATGTTGTC-3' | 110 | NM_016674.4 |
